# Phosphorus remobilization from rice flag leaves during grain filling: an RNA‐seq study

**DOI:** 10.1111/pbi.12586

**Published:** 2016-06-27

**Authors:** Kwanho Jeong, Abdul Baten, Daniel L. E. Waters, Omar Pantoja, Cecile C. Julia, Matthias Wissuwa, Sigrid Heuer, Tobias Kretzschmar, Terry J. Rose

**Affiliations:** ^1^ Southern Cross Plant Science Southern Cross University Lismore NSW Australia; ^2^ Southern Cross GeoScience Southern Cross University Lismore NSW Australia; ^3^ Instituto de Biotecnología Universidad Nacional Autónoma de México Cuernavaca Morelos Mexico; ^4^ Crop, Livestock and Environment Division Japan International Research Center for Agricultural Sciences Tsukuba Ibaraki Japan; ^5^ University of Adelaide School of Agriculture Food and Wine / Australian Centre for Plant Functional Genomics (ACPFG) Adelaide SA Australia; ^6^ Genotyping Services Laboratory International Rice Research Institute (IRRI) Metro Manila Philippines

**Keywords:** Illumina sequencing, *Oryza sativa*, differential gene expression, phosphorus translocation, senescence

## Abstract

The physiology and molecular regulation of phosphorus (P) remobilization from vegetative tissues to grains during grain filling is poorly understood, despite the pivotal role it plays in the global P cycle. To test the hypothesis that a subset of genes involved in the P starvation response are involved in remobilization of P from flag leaves to developing grains, we conducted an RNA‐seq analysis of rice flag leaves during the preremobilization phase (6 DAA) and when the leaves were acting as a P source (15 DAA). Several genes that respond to phosphate starvation, including three purple acid phosphatases (*OsPAP3, OsPAP9b* and *OsPAP10a*), were significantly up‐regulated at 15 DAA, consistent with a role in remobilization of P from flag leaves during grain filling. A number of genes that have not been implicated in the phosphate starvation response, *OsPAP26, SPX‐MFS1* (a putative P transporter) and *SPX‐MFS2*, also showed expression profiles consistent with involvement in P remobilization from senescing flag leaves. Metabolic pathway analysis using the KEGG system suggested plastid membrane lipid synthesis is a critical process during the P remobilization phase. In particular, the up‐regulation of *OsPLDz2* and *OsSQD2* at 15 DAA suggested phospholipids were being degraded and replaced by other lipids to enable continued cellular function while liberating P for export to developing grains. Three genes associated with RNA degradation that have not previously been implicated in the P starvation response also showed expression profiles consistent with a role in P mobilization from senescing flag leaves.

## Introduction

Senescence is the final stage of plant development (Kong *et al*., 2013). It is typically characterized by visible leaf yellowing and is controlled by both environmental cues and internal elements such as phytohormones and gene regulators (Breeze *et al*., 2011; Dong *et al*., 2012). A range of biotic and abiotic environmental cues have been implicated in inducing senescence, including pathogen infection, drought, nutrition limitation and oxidative stress (Buchanan‐Wollaston *et al*., 2003). The yellowing of senescing leaves is related to the degradation of macromolecules like chlorophyll, proteins and RNA, and this degradation ultimately results in a decline in photosynthetic activity (Buchanan‐Wollaston, 1997; Guo *et al*., 2004).

Another key component of senescence in annual crop plants is the remobilization of mineral nutrients from vegetative organs to seeds (Kong *et al*., 2015). Phosphorus (P) is remobilized to developing seeds with particularly high efficiency, resulting in P harvest indices well above the carbon harvest indices in cultivars of most major crop species (Araújo and Teixeira, 2003; Batten, 1992; Rose *et al*., 2007, 2010). Although this may be ecologically advantageous because high seed P content improves seedling competitiveness in soils that are naturally low in bioavailable P (White and Veneklaas, 2012), in the context of agriculture it results in the removal of vast amounts of P from fields in harvested products.

The loading of P into grains is of critical importance for the global P cycle because the accumulation of P in the grains of the major cereals, oilseed and pulse crops globally removes the equivalent of over 50% of the P applied as fertilizer each year (Lott *et al*., 2000). Provided seed germination and seedling vigour can be maintained (see Pariasca‐Tanaka *et al*., 2015 and discussion therein), reducing the amount of P stored in the grains of major crops may be a viable option to reduce the amount of P lost from the P cycle within agricultural production systems (Raboy, 2009; Rose and Wissuwa, 2012; Rose *et al*., 2013). Although breeding crop cultivars with reduced P levels in grains is an attractive solution, practical advances are hampered by our limited understanding of the physiology, and genetic and molecular regulation of P loading into developing grains, including P remobilization from senescing vegetative tissues (Wang *et al*., 2016).

The degradation of lipids, nucleic acids and proteins is a key component of the senescence process, and it is possible some of the genes involved in recycling P from phospholipids, RNA and proteins under P starvation in vegetative growth may also play a role in the mobilization of P from senescing tissues, as discussed in a number of recent reviews (Smith *et al*., 2015; Stigter and Plaxton, 2015). However, to the best of our knowledge, the only genes that have been definitively linked to P loading in developing grains are a putative sulphate transporter which has an endosperm‐specific impact on grain P in barley (Raboy *et al*., 2014; Ye *et al*., 2011), the ATP‐binding cassette (ABC) transporters *ZmMRP4* and *AtMPR5/AtABCC5* which, when inactive, result in low seed P in maize and *Arabidopsis*, respectively (Nagy *et al*., 2009; Shi *et al*., 2007), and the purple acid phosphatase (PAP) *AtPAP26* which is involved in remobilization of P from senescing leaves to developing seeds in *Arabidopsis* (Robinson *et al*., 2012). NAC transcription factors are also involved in nutrient remobilization during senescence (Stigter and Plaxton, 2015); for example, the NAC transcription factor *NAM‐B1* plays a role in P loading into wheat grains, but it appears to play a broad role in nutrient remobilization to grains not limited to P (Uauy *et al*., 2006).

Given the key role of gene families such as P transporters (PTs), PAPs and SPX domain proteins (named after proteins SYG1/PHO81/XPR1) in P recycling under P starvation stress (Wang *et al*., 2012; Wu *et al*., 2013), and the role of RNases in remobilization of P from senescing hakea leaves (Shane *et al*., 2014), we hypothesized some members of these gene families may be involved in the remobilization of P from senescing leaves during grain filling. To test this hypothesis, we first identified a total of 115 P starvation‐related (PSR) genes based on recent literature (Lin *et al*., 2009; Liu *et al*., 2011; MacIntosh *et al*., 2010; Secco *et al*., 2013) (see Table S1). We then investigated the expression of these genes in flag leaves during grain filling in the model cereal rice (*Oryza sativa* L.) using an RNA sequencing (RNA‐seq) approach.

Several studies have investigated gene transcript abundance during leaf senescence in crops such as cotton and maize (Lin *et al*., 2015; Zhang *et al*., 2014) and in a range tissues during grain filling in rice (e.g. Duan and Sun, 2005; Zhu *et al*., 2003), including flag leaf tissue, and transcript data are publically available. However, without concurrent data on tissue P dynamics, it is not possible to put these data in context. Here, we quantified P dynamics in flag leaves during grain filling and subsequently examined gene expression at key time points when P concentrations in the flag leaf were stable compared with when the flag leaf was acting as a P source.

## Results

### Remobilization of phosphorus from flag leaves during grain filling

**Figure 1:**
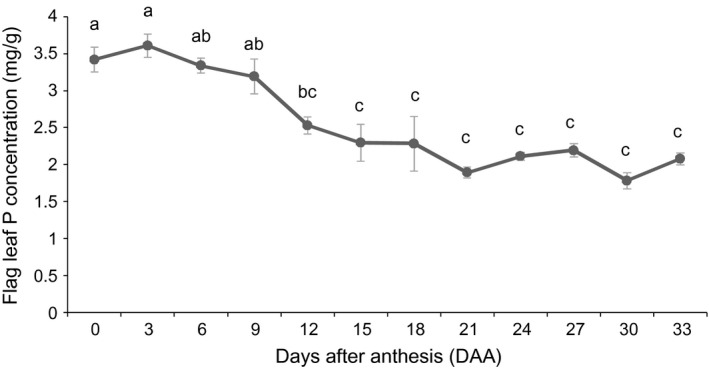
Phosphorus concentration of flag leaves during grain filling. Each value represents the mean ± SE of three biological replicates. Different letters indicate significant difference at *P* ≤ 0.01.

Flag leaf P concentrations were relatively stable during early grain filling, but declined significantly from 9 days after anthesis (DAA) (Figure 1). The decline in leaf P concentration from around 3.5 mg/g at anthesis to around 2.5 mg/g at 12 DAA suggests the leaf was acting as a ‘P source’ by 12 DAA. Two time points were selected for the RNA‐seq study: 6 DAA, when the P concentrations were stable (flag leaf was not acting as a P source), and 15 DAA, when the flag leaf was clearly acting as a P source as shown by a 33% reduction in P concentration relative to 0 DAA. The 15 DAA time point was selected because it was the first time point that had a significantly lower P concentration than the 6 DAA time point.

### Global gene expression of RNA‐seq data

**Figure 2:**
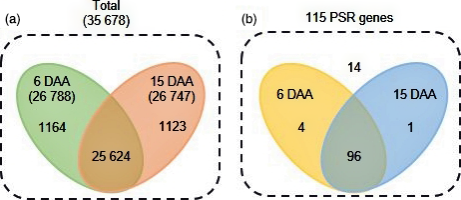
Venn diagram showing (a) the total number of genes expressed between two time points and (b) the expression profile of 115 PSR genes.

Three biological replicates for each time point (6 DAA and 15 DAA) were analysed using RNA‐seq. These six libraries generated 156 million 101‐bp paired‐end reads after quality control using FASTQC software; 83 million and 73 million from 6 DAA and 15 DAA, respectively (Table 1). Reads were filtered for adapter sequences, poly‐N stretches and low‐quality reads which resulted in 78 million (94%) and 70 million (95.6%) high‐quality reads from 6 DAA and 15 DAA, respectively (Table 1). Of these high‐quality reads, around 60 million (77.5%) from 6 DAA and 56 million (79.7%) from 15 DAA were mapped to the reference genome (Table 1). There were 26 788 expressed genes at 6 DAA and 26 747 at 15 DAA (Figure 2a) and 5160 differentially expressed genes (DEGs) between the two time points using a cut‐off based on *P* < 0.05 and false discovery rate (FDR) < 0.05 values.

**Table 1:** General information of sequencing reads and mapping statistics

Samples	Replications	Raw reads	High‐quality reads		Mapping to genome	
			Number	%	Number	%
6 DAA	1	30 160 726	28 586 476	94.78	22 181 542	77.59
	2	27 979 092	25 551 322	91.32	19 050 896	74.56
	3	25 297 097	24 329 299	96.17	19 612 039	80.61
15 DAA	1	22 787 486	21 747 029	95.43	17 210 696	79.14
	2	25 016 582	23 801 938	95.14	18 800 985	78.99
	3	25 603 135	24 652 300	96.29	19 961 618	80.97

### GO enrichment analysis of DEGs

To classify the function of DEGs between two time points, gene ontology (GO) enrichment analysis was performed looking at DEGs at 15 DAA relative to 6 DAA. After filtering for DEGs based on *P* and FDR values (i.e. <0.05) as well as log_2_ fold change (i.e. >1.5), 1643 DEGs were retained. Using parametric analysis of gene set enrichment (PAGE), 1180 DEGs were mapped to the GO term database resulting in a total of 263 enriched GO terms.

**Figure 3:**
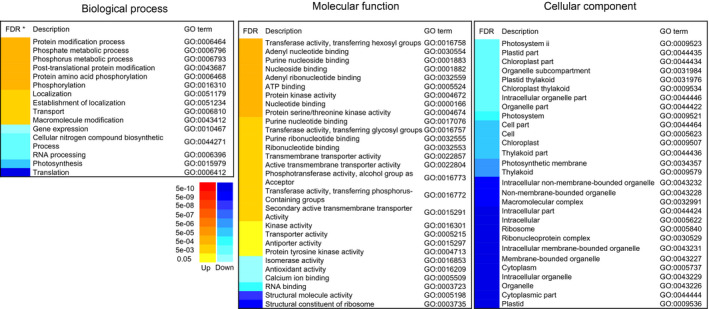
Hierarchical clustering of 72 significant GO terms by PAGE analysis. The adjusted FDR value of the term determines the degree of colour saturation of the corresponding box.

Up‐regulated DEGs within the term biological process were largely associated with P metabolism, protein modification and transport, which was congruent with up‐regulated DEGs within the molecular function term that were found to be associated with phosphotransferase, kinase or transport activity. Interestingly, a high number of up‐regulated DEGs could be categorized to nine GO terms related to nucleotide, nucleoside or ribonucleotide binding proteins, although the genes found in each of the respective GO terms are largely identical. No up‐regulated DEGs were enriched within the cellular component GO term (Figure 3). Down‐regulated DEGS within biological processes were associated with transcription and translation, while, congruently, down‐regulated DEGs under molecular functions were associated with RNA binding and ribosome constitution. Several genes related to photosynthesis and photosystems were down‐regulated, which was further reflected in a marked down‐regulation of cellular component genes associated with plastids and organelles.

### Expression of 115 PSR genes in the flag leaf at 6 DAA and 15 DAA

Of the 115 PSR genes, 100 were expressed at 6 DAA and 97 at 15 DAA in flag leaves (Figure 2b). The most highly expressed gene of the 115 PSR genes was the P transporter (*OsPT*) *OsPT21* (Os01g0279700) with expression levels of 1613 FPKM (fragments per kilobase of transcript per million mapped reads) at 6 DAA and 1168 FPKM at 15 DAA. The second most highly expressed of the 115 PSR genes was *OsPT24* (Os09g0570400) with 694 FPKM at 6 DAA and 334 FPKM at 15 DAA (Table S1). Only 14 of the 115 PSR genes were not expressed (FPKM = 0) at either 6 DAA or 15 DAA (Figure 2b).

We identified 38 PSR genes that were differentially expressed between the two time points (Table 2). Twenty‐six of the 38 genes were up‐regulated at 15 DAA when the flag leaf acts as a source of P for developing grains (Table 2). These 26 genes included *OsPHO1* and *OsPHO2,* which are primary factors in P starvation signalling, as well as *OsSPX1* and *OsSPX2* domain proteins that play a key a role in P homeostasis during vegetative growth (Secco *et al*., 2012; Wu *et al*., 2013). Further, three PAPs (*OsPAP1d*,* OsPAP3c* and *OsPAP10a*) which are involved in the release of inorganic P (Pi) from organic P (Wu *et al*., 2013) and *OsSQD2* which is involved in sugar transport to diacylglycerol (DAG) were among the 26 genes. Three of the 26 known P transporters (Liu *et al*., 2011), *OsPT5*,* OsPT19* and *OsPT20,* were also among the 26 PSR genes up‐regulated at 15 DAA in the flag leaf (Table 2).

**Table 2:** 38 differentially expressed genes among 115 PSR genes

No.	Gene id	MSU id	Name/description	FPKM		Log_2_
				6 DAA	15 DAA	
1	OS07G0187400	LOC_Os07g08970	Conserved hypothetical protein	44.01	288.27	2.71
2	OS03G0238600	LOC_Os03g13540	*OsPAP3c*	10.74	59.36	2.47
3	OS07G0165200	LOC_Os07g07080	Regulator of chromosome condensation/beta‐lactamase‐inhibitor protein II	8.59	35.30	2.04
4	OS02G0514500	LOC_Os02g31030	Glycerophosphoryl diester phosphodiesterase family protein	6.06	24.77	2.03
5	OS06G0291500	LOC_Os06g18820	Conserved hypothetical protein.	0.34	1.36	1.99
6	OS01G0142300	LOC_Os01g04920	*OsSQD2*	12.03	37.18	1.63
7	OS04G0185600	LOC_Os04g10690	*OsPT5*	0.30	0.90	1.60
8	OS01G0110100	LOC_Os01g02000	*OsPHO1;1*	2.50	7.30	1.55
9	OS02G0802700	LOC_Os02g55910	Similar to MGDG synthase type A	12.18	34.87	1.52
10	OS09G0554000	LOC_Os09g38100	*OsPT20*	1.80	5.00	1.47
11	OS03G0261800	LOC_Os03g15530	Protein of unknown function DUF3049 domain containing protein	12.71	33.58	1.40
12	OS01G0310100	LOC_Os01g20860	*OsPLDzeta2, OsPLDrho2*	3.35	8.74	1.38
13	OS01G0776600	LOC_Os01g56880	*OsPAP10a*	16.63	41.37	1.31
14	OS05G0557700	LOC_Os05g48390	*OsPHO2*	8.93	18.89	1.08
15	OS07G0134500	LOC_Os07g04210	Similar to hydrolase/protein serine/threonine phosphatase	9.82	20.74	1.08
16	OS08G0156600	LOC_Os08g06010	Major facilitator superfamily protein.	8.95	18.34	1.03
17	OS01G0557500	LOC_Os01g37690	*OsCAX1a*	19.79	38.98	0.98
18	OS03G0214400	LOC_Os03g11560	Digalactosyldiacylglycerol synthase, chloroplast precursor	5.74	11.04	0.94
19	OS09G0454600	LOC_Os09g28160	*OsPT19*	3.56	6.68	0.91
20	OS05G0358700	LOC_Os05g29050	*OsPLDrho1*	9.97	16.72	0.75
21	OS09G0528700	LOC_Os09g35940	Similar to Cytochrome p450 (CYP78A9).	15.78	26.39	0.74
22	OS06G0140800	LOC_Os06g04880	Serine threonine kinase, putative	24.09	39.60	0.72
23	OS06G0603600	LOC_Os06g40120	*OsSPX1*	31.50	51.15	0.70
24	OS02G0202200	LOC_Os02g10780	*OsSPX2*	33.67	54.16	0.69
25	OS12G0576600	LOC_Os12g38750	*OsPAP1d*	12.11	18.76	0.63
26	OS08G0433200	LOC_Os08g33640	Conserved hypothetical protein.	34.26	50.60	0.56
27	OS09G0537700	LOC_Os09g36680	*OsRNS4*	0.72	0.00	Undefined^a^
28	OS02G0625300	LOC_Os02g41580	CAMK_CAMK_like.14 – CAMK includes calcium/calmodulin depedent protein kinases	38.41	25.68	−0.58
29	OS04G0608600	LOC_Os04g51920	Protein disulfide isomerase	14.28	9.11	−0.65
30	OS07G0100300	LOC_Os07g01030	Glycosyl transferase, group 1 domain containing protein	131.17	80.56	−0.70
31	OS04G0598000	LOC_Os04g50970	Seed specific protein Bn15D1B	553.88	297.77	−0.90
32	OS10G0444700	LOC_Os10g30790	*OsPT8*	13.26	7.11	−0.90
33	OS02G0593500	LOC_Os02g38020	*OsPT14*	35.75	16.15	−1.15
34	OS07G0187700	LOC_Os07g09000	*OsPHF1*	28.30	12.13	−1.22
35	OS03G0263400	LOC_Os03g15690	*OsPT16*	11.83	5.05	−1.23
36	OS01G0852200	LOC_Os01g63290	*OsPT22*	49.99	18.59	−1.43
37	OS02G0668500	LOC_Os02g44820	Cellular retinaldehyde‐binding/triple function,C‐terminal domain containing protein	35.42	12.24	−1.53
38	OS05G0387200	LOC_Os05g32140	Similar to UDP‐sulfoquinovose synthase,chloroplast precursor (EC 3. 13. 1. 1)	219.30	60.06	−1.87

All the genes were cut off by *P* < 0.05 and FDR < 0.05.

a Undefined = zero expression value for one of the two samples.

### Expression of *OsPAP*,* OsPT* and *OsSPX* domain gene families in the flag leaf at 6 DAA and 15 DAA

**Figure 4:**
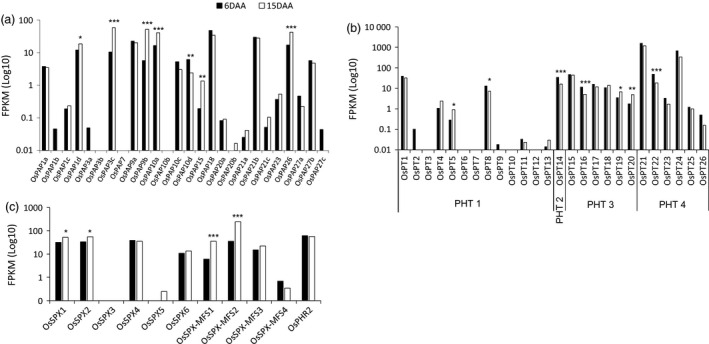
The expression of (a) 26 *OsPAPs*, (b) 26 *OsPTs* and (c) 6 *OsSPXs and 4 OsSPX‐MFS* domains and their regulatory transcription factor *OsPHR2* in the flag leaf during grain filling in rice. ***P < 0.001; **P < 0.01, *P < 0.05; no mark = nonsignificant.

Given that several genes in the *OsPT*,* OsPAP* and *OsSPX* gene families were differentially expressed between 6 DAA and 15 DAA, we investigated expression of all known members of these gene families at both time points. Of the 26 *OsPAP* genes in rice (Zhang *et al*., 2011), in addition to the three *OsPAP* genes included in the 115 PSR gene*s* that were up‐regulated at 15 DAA, a further three *OsPAP* genes (*OsPAP9b*,* OsPAP15* and *OsPAP26*) had significantly up‐regulated expression (log_2_ fold change >1.3) at 15 DAA, although the expression of *OsPAP15* was low compared to the other *OsPAP* genes (Figure 4a).

Twenty‐one of the 26 P transporters in rice (*OsPT1* to *OsPT26*) were expressed in flag leaves. Three of these—*OsPT5*,* OsPT19* and *OsPT20*—were differentially expressed at 15 DAA (Figure 4b). Expression levels of *OsPT19* and *OsPT20* were relatively low, and the expression level of *OsPT5* was extremely low (Figure 4b). Interestingly, while *OsPT21* and *OsPT24* were not significantly differentially expressed between the time points, their overall expression was the highest (>400 FKPM) at both 6 DAA and 15 DAA (Figure 4b).

Six *SPX* genes (*OsSPX1‐OsSPX6*) (Wang *et al*., 2009) and four *SPX‐MFS* (major facility superfamily) genes (*OsSPX‐MFS1‐4*) are known to be regulated by *OsPHR2* under P deficiency in rice (Lin *et al*., 2010; Wang *et al*., 2012; Wu *et al*., 2013). Examination of all *OsSPX* and *OsSPX‐MFS* expression levels and their regulator *OsPHR2* revealed that in addition to *OsSPX1* and *OsSPX2* (see above), *OsSPX*‐*MFS1* and *OsSPX*‐*MFS2* were also significantly up‐regulated at 15 DAA with log_2_ fold changes of 2.55 and 2.80, respectively. The absolute expression level of *OsSPX‐MFS2* was greater than 200 FPKM at 15 DAA (Figure 4c). Interestingly, expression of the transcription factor *OsPHR2* was not significantly up‐regulated at 15 DAA.

### Biological pathways related with P recycling processes during senescence

To investigate the possible involvement of lipid and nucleotide metabolism genes in P remobilization during leaf senescence, we used the KEGG pathway database to retrieve genes associated with these metabolic pathways. We then analysed expression of all DEGs in our RNA‐seq data and found that 35 DEGs mapped to lipid metabolism (Table 3). Among these, glycerophosphoryl diester phosphodiesterase (GDPD; PLC‐like; Os02g0514500), digalactosyldiacylglycerol (DGDG) synthase (Os02g0539100), monogalactosyldiacylglycerol (MGDG) synthase (Os02G0802700) and sulphoquinovosyltransferase (*SQD2;* Os01G0142300), whose products have been proposed to participate in galactolipid synthesis, were up‐regulated at 15 DAA (Table 3). Several genes related to lipid degradation were also up‐regulated, such as phospholipase D (Os06g0604400) and phospholipase C (Os03g0826600), glycerophosphoryl diester phosphodiesterase (Os02g0514500), aldehyde dehydrogenase (NAD^+^) (Os12g0178000) and phosphatidate phosphatase (Os05g0462400). In contrast, dihydroxyacetone kinase (Os03g0719300), glycerol‐3‐phosphate dehydrogenase (NAD^+^) (Os01g0971600, Os07g0229800), glycerol‐3‐phosphate O‐acyltransferase (Os10g0577900) involved in *de novo* DAG synthesis were down‐regulated (Table 3). Also down‐regulated were lysophospholipid acyltransferase (Os02g0676000) and 1‐acyl‐sn‐glycerol‐3‐phosphate acyltransferase (Os10g0497100), gene products involved in phospholipid synthesis.

**Table 3:** Differentially expressed genes associated with lipid metabolic pathways

No.	Gene id	KEGG Number and description	FPKM		Log_2_
			6 DAA	15 DAA	
1	Os02g0514500	K18696 GDE1; glycerophosphoryl diester phosphodiesterase [EC:3.1.4.46]	6.06	24.77	2.03
2	Os12g0178000	K00128 E1.2.1.3; aldehyde dehydrogenase (NAD^+^) [EC:1.2.1.3]	1.80	7.29	2.02
3	Os05g0462400	K15728 LPIN; phosphatidate phosphatase LPIN [EC:3.1.3.4]	4.11	15.08	1.88
4	Os10g0473400	K17108 GBA2; nonlysosomal glucosylceramidase [EC:3.2.1.45]	3.36	11.28	1.75
5	Os01g0142300	K06119 SQD2; sulfoquinovosyltransferase [EC:2.4.1.‐]	12.03	37.18	1.63
6	Os02g0802700	K03715 E2.4.1.46; 1,2‐diacylglycerol 3‐beta‐galactosyltransferase [EC:2.4.1.46]	12.18	34.87	1.52
7	Os02g0539100	K09480 E2.4.1.241; digalactosyldiacylglycerol synthase [EC:2.4.1.241]	30.23	80.43	1.41
8	Os06g0204400	K00993 EPT1; ethanolaminephosphotransferase [EC:2.7.8.1]	25.77	52.27	1.02
9	Os11g0158400	K09480 E2.4.1.241; digalactosyldiacylglycerol synthase [EC:2.4.1.241]	9.40	18.58	0.98
10	Os03g0214400	K09480 E2.4.1.241; digalactosyldiacylglycerol synthase [EC:2.4.1.241]	5.74	11.04	0.94
11	Os06g0604400	K01115 PLD1_2; phospholipase D1/2 [EC:3.1.4.4]	7.92	15.06	0.93
12	Os11g0242100	K17108 GBA2; nonlysosomal glucosylceramidase [EC:3.2.1.45]	19.35	36.26	0.91
13	Os03g0826600	K01114 plcC; phospholipase C [EC:3.1.4.3]	166.74	303.14	0.86
14	Os12g0121300	K00967 PCYT2; ethanolamine‐phosphate cytidylyltransferase [EC:2.7.7.14]	4.83	8.19	0.76
15	Os01g0624000	K12349 ASAH2; neutral ceramidase [EC:3.5.1.23]	58.55	98.81	0.75
16	Os04g0634700	K00901 dgkA; diacylglycerol kinase (ATP) [EC:2.7.1.107]	16.18	26.75	0.73
17	Os11g0186200	K00128 E1.2.1.3; aldehyde dehydrogenase (NAD^+^) [EC:1.2.1.3]	283.39	464.74	0.71
18	Os11g0516000	K00654 SPT; serine palmitoyltransferase [EC:2.3.1.50]	43.67	69.55	0.67
19	Os01g0931300	K13511 TAZ; monolysocardiolipin acyltransferase [EC:2.3.1.‐]	32.54	51.62	0.67
20	Os08g0224800	K00967 PCYT2; ethanolamine‐phosphate cytidylyltransferase [EC:2.7.7.14]	22.09	34.10	0.63
21	Os02g0676000	K13519 LPT1; lysophospholipid acyltransferase [EC:2.3.1.51 2.3.1.23 2.3.1.‐]	95.28	59.76	−0.67
22	Os07g0100300	K06119 SQD2; sulfoquinovosyltransferase [EC:2.4.1.‐]	131.17	80.56	−0.70
23	Os10g0497100	K00655 plsC; 1‐acyl‐sn‐glycerol‐3‐phosphate acyltransferase [EC:2.3.1.51]	22.15	13.38	−0.73
24	Os01g0175000	K06130 LYPLA2; lysophospholipase II [EC:3.1.1.5]	51.15	30.17	−0.76
25	Os04g0669500	K06130 LYPLA2; lysophospholipase II [EC:3.1.1.5]	21.69	12.41	−0.81
26	Os05g0548900	K05929 E2.1.1.103; phosphoethanolamine N‐methyltransferase [EC:2.1.1.103]	18.11	9.72	−0.90
27	Os01g0796500	K01094 GEP4; phosphatidylglycerophosphatase GEP4 [EC:3.1.3.27]	36.87	19.52	−0.92
28	Os06g0649900	K16860 PLD3_4; phospholipase D3/4 [EC:3.1.4.4]	3.63	1.88	−0.95
29	Os03g0719300	K00863 E2.7.1.29; dihydroxyacetone kinase [EC:2.7.1.29]	46.29	21.50	−1.11
30	Os10g0577900	K00630 ATS1; glycerol‐3‐phosphate O‐acyltransferase [EC:2.3.1.15]	56.31	25.82	−1.13
31	Os01g0971600	K00006 GPD1; glycerol‐3‐phosphate dehydrogenase (NAD^+^) [EC:1.1.1.8]	4.87	1.75	−1.47
32	Os10g0493600	K07407 E3.2.1.22B; alpha‐galactosidase [EC:3.2.1.22]	173.12	61.63	−1.49
33	Os07g0229800	K00006 GPD1; glycerol‐3‐phosphate dehydrogenase (NAD^+^) [EC:1.1.1.8]	42.68	14.48	−1.56
34	Os05g0387200	K06118 SQD1; UDP‐sulfoquinovose synthase [EC:3.13.1.1]	219.30	60.06	−1.87
35	Os07g0679300	K07407 E3.2.1.22B; alpha‐galactosidase [EC:3.2.1.22]	27.76	5.94	−2.23

We also examined the nucleotide metabolism pathway that includes RNA transport and degradation and found 50 DEGs, of which 16 genes were up‐regulated and 34 genes were down‐regulated at 15 DAA (Table 4). Among the up‐regulated genes, six are directly related to RNA degradation (numbers 1, 2, 9, 13, 14 and 15 in Table 4). In general, the down‐regulated genes within the nucleotide metabolism pathway are involved in nucleic acid biosynthesis/maturation/processing (19, 26, 33, 36, 38, 39, 41, 44, 47‐51; Table 4) or purine biosynthesis (17, 21, 29, 40, 42 and 43; Table 4), which, together with the up‐regulated genes, indicates a strong degradation of nucleic acids during leaf senescence.

**Table 4:** Differentially expressed genes associated with nucleotide metabolic pathways

No.	Gene id	KEGG Number and description	FPKM		Log_2_
			6 DAA	15 DAA	
1	Os10g0556700	K12604 CNOT1; CCR4‐NOT transcription complex subunit 1	2.20	7.94	1.85
2	Os10g0556600	K12604 CNOT1; CCR4‐NOT transcription complex subunit 1	6.46	19.83	1.62
3	Os03g0743900	K13811 PAPSS; 3′‐phosphoadenosine 5′‐phosphosulfate synthase [EC:2.7.7.4 2.7.1.25]	22.79	67.60	1.57
4	Os07g0661000	K01488 add; adenosine deaminase [EC:3.5.4.4]	7.10	19.48	1.46
5	Os05g0151000	K03006 RPB1; DNA‐directed RNA polymerase II subunit RPB1 [EC:2.7.7.6]	0.40	1.03	1.37
6	Os04g0680400	K01466 allB; allantoinase [EC:3.5.2.5]	20.50	51.62	1.33
7	Os04g0111200	K13811 PAPSS; 3′‐phosphoadenosine 5′‐phosphosulfate synthase [EC:2.7.7.4 2.7.1.25]	35.63	87.25	1.29
8	Os06g0151900	K00850 pfkA; 6‐phosphofructokinase 1 [EC:2.7.1.11]	16.82	32.47	0.95
9	Os07g0249600	K12611 DCP1B; mRNA‐decapping enzyme 1B [EC:3.‐.‐.‐]	10.47	19.87	0.92
10	Os08g0175600	K02335 DPO1; DNA polymerase I [EC:2.7.7.7]	6.01	11.22	0.90
11	Os11g0148500	K00873 PK; pyruvate kinase [EC:2.7.1.40]	72.24	128.11	0.83
12	Os08g0109300	K00939 adk; adenylate kinase [EC:2.7.4.3]	115.98	197.90	0.77
13	Os03g0316900	K12608 CAF16; CCR4‐NOT complex subunit CAF16	8.36	14.02	0.75
14	Os03g0748800	K14442 DHX36; ATP‐dependent RNA helicase DHX36 [EC:3.6.4.13]	8.84	14.64	0.73
15	Os01g0256900	K12623 LSM4; U6 snRNA‐associated Sm‐like protein LSm4	28.87	43.89	0.60
16	Os04g0677500	K00873 PK; pyruvate kinase [EC:2.7.1.40]	56.13	82.28	0.55
17	Os03g0313600	K01756 purB; adenylosuccinate lyase [EC:4.3.2.2]	51.84	34.23	−0.60
18	Os12g0589100	K00759 APRT; adenine phosphoribosyltransferase [EC:2.4.2.7]	146.48	96.30	−0.61
19	Os04g0428950	K14525 RPP25; ribonucleases P/MRP protein subunit RPP25 [EC:3.1.26.5]	69.88	45.16	−0.63
20	Os03g0831500	K01933 purM; phosphoribosylformylglycinamidine cyclo‐ligase [EC:6.3.3.1]	18.98	11.83	−0.68
21	Os05g0389300	K12624 LSM5; U6 snRNA‐associated Sm‐like protein LSm5	64.94	39.91	−0.70
22	Os05g0389500	K12603 CNOT6; CCR4‐NOT transcription complex subunit 6 [EC:3.1.13.4]	53.51	32.55	−0.72
23	Os08g0270200	K12592 C1D; exosome complex protein LRP1	28.37	16.60	−0.77
24	Os07g0495000	K14977 ylbA; (S)‐ureidoglycine aminohydrolase [EC:3.5.3.26]	46.29	26.69	−0.79
25	Os02g0168900	K10745 RNASEH2C; ribonuclease H2 subunit C	14.48	8.31	−0.80
26	Os04g0443900	K04077 groEL; chaperonin GroEL	434.11	239.14	−0.86
27	Os03g0320900	K00942 E2.7.4.8; guanylate kinase [EC:2.7.4.8]	25.64	13.99	−0.87
28	Os08g0206600	K00602 purH; phosphoribosylaminoimidazolecarboxamide formyltransferase/IMP cyclohydrolase [EC:2.1.2.3 3.5.4.10]	34.51	18.77	−0.88
29	Os07g0168000	K00962 pnp; polyribonucleotide nucleotidyltransferase [EC:2.7.7.8]	80.00	42.30	−0.92
30	Os03g0780500	K00088 guaB; IMP dehydrogenase [EC:1.1.1.205]	92.09	47.95	−0.94
31	Os01g0865100	K00365 uaZ; urate oxidase [EC:1.7.3.3]	37.21	19.36	−0.94
32	Os06g0168600	K10807 RRM1; ribonucleoside‐diphosphate reductase subunit M1 [EC:1.17.4.1]	5.53	2.75	−1.01
33	Os03g0130400	K00939 adk; adenylate kinase [EC:2.7.4.3]	126.52	62.57	−1.02
34	Os05g0349200	K01490 AMPD; AMP deaminase [EC:3.5.4.6]	65.88	32.58	−1.02
35	Os05g0595400	K00940 ndk; nucleoside‐diphosphate kinase [EC:2.7.4.6]	84.30	41.54	−1.02
36	Os07g0611600	K03024 RPC7; DNA‐directed RNA polymerase III subunit RPC7	17.52	8.47	−1.05
37	Os06g0257450	K10808 RRM2; ribonucleoside‐diphosphate reductase subunit M2 [EC:1.17.4.1]	10.51	5.08	−1.05
38	Os03g0844450	K12587 MTR3; exosome complex component MTR3	10.41	4.91	−1.08
39	Os05g0270800	K00601 E2.1.2.2; phosphoribosylglycinamide formyltransferase [EC:2.1.2.2]	9.31	4.25	−1.13
40	Os04g0388900	K12625 LSM6; U6 snRNA‐associated Sm‐like protein LSm6	17.50	7.50	−1.22
41	Os01g0888500	K01952 purL; phosphoribosylformylglycinamidine synthase [EC:6.3.5.3]	64.76	27.15	−1.25
42	Os05g0430800	K00764 purF; amidophosphoribosyltransferase [EC:2.4.2.14]	5.86	2.44	−1.26
43	Os04g0692500	K18213 PRORP; proteinaceous RNase P [EC:3.1.26.5]	35.63	14.75	−1.27
44	Os10g0457500	K01519 ITPA; inosine triphosphate pyrophosphatase [EC:3.6.1.19]	10.45	4.16	−1.33
45	Os04g0684900	K12581 CNOT7_8; CCR4‐NOT transcription complex subunit 7/8	2.03	0.79	−1.36
46	Os03g0699300	K01939 purA; adenylosuccinate synthase [EC:6.3.4.4]	41.47	16.12	−1.36
47	Os09g0482680	K00784 rnz; ribonuclease Z [EC:3.1.26.11]	138.72	46.97	−1.56
48	Os08g0116800	K12587 MTR3; exosome complex component MTR3	3.64	0.93	−1.97
49	Os02g0273800	K18213 PRORP; proteinaceous RNase P [EC:3.1.26.5]	79.60	15.86	−2.33
50	Os12g0548300	K00940 ndk; nucleoside‐diphosphate kinase [EC:2.7.4.6]	38.89	7.56	−2.36

## Discussion

While previous studies have profiled gene expression in a range of tissues during rice grain filling (e.g. Duan and Sun, 2005; Zhu *et al*., 2003) and leaf senescence in other crops such as cotton (Lin *et al*., 2015) and maize (Zhang *et al*., 2014), lack of concurrent data on P remobilization precludes conclusions being made about the potential regulation of P remobilization from vegetative tissues during grain filling. In the present study, we identified critical developmental stages during grain filling when flag leaf P concentration was stable (6 DAA) and when the flag leaf was a P source (15 DAA) as the basis for subsequent RNA‐seq studies.

Given the dearth of knowledge on specific genes involved in P remobilization from senescing leaves during grain filling (Wang *et al*., 2016), we investigated whether genes associated with P starvation during vegetative growth were also associated with P remobilization from vegetative tissues during grain filling. Of the 115 PSR genes identified in the published literature, 14 were not expressed in flag leaves at either developmental stage, which is not surprising given that some of these genes have tissue‐specific expression. *OsRNS1* and *OsRNS7,* for example, are specifically expressed in root tissue (MacIntosh *et al*., 2010). As expected, the absolute expression of the 115 PSR genes was low relative to the most highly expressed genes as many of the highly expressed genes (e.g. *OsLIR1*, photosystem subunits I, II and chloroplast precursor) are key genes for photosynthesis and are highly expressed in flag leaves during grain filling (Reimmann and Dudler, 1993; Sugano *et al*., 2010).

GO enrichment analysis is a common and powerful approach in gene expression analysis (Subramanian *et al*., 2005). By categorizing treatment or development‐dependent global transcript changes into standardized GO terminology, metabolic shifts are revealed, allowing for the interpretation of possible changes that may occur at the molecular and cellular level (Du *et al*., 2010). Down‐regulated DEGs were highly enriched in GO terms related to photosynthesis (GO:0015979) and plastids (45% of the cellular component category), indicating a reduction of photo‐assimilatory anabolism at 15 DAA. Furthermore, down‐regulated DEGs were enriched in GO terms related to transcription and translation (GO:0010467, GO:0006396, GO:0003723, GO:0003735, GO:0006412, GO:0005840 and GO:00529), while up‐regulated genes were enriched in GO terms related to protein modification (GO:0006464 and GO:0043687) and protein phosphorylation (GO:0006468, GO:0004672, GO:0004674, GO:0016301 and GO:0004713). This suggests that at 15 DAA, regulation of metabolism was achieved through the modulation of existing proteins rather than synthesis of new proteins, which is consistent with a change from anabolism towards catabolism and the remobilization of nutrients at 15 DAA. Resources are recycled and removed rather than re‐invested within the flag leaf, which is supported by the finding that up‐regulated DEGs were enriched in GO terms related to transport (GO:0006810, GO:0022857, GO:0022804, GO:0015291, GO:0005215, GO:0015297). Collectively, this clearly indicates a shift towards senescence at 15 DAA.

Markedly enriched up‐regulated DEGs were found in GO terms related to P metabolism (GO:0006796, GO:0006793, GO:0016310, GO:0016772 and GO:0016773), which corresponds to the observation of enhanced P remobilization from flag leaves at 15 DAA. However, none of the identified 115 PSR genes were found among the enriched genes within P metabolism‐related GO terms. We therefore employed a targeted approach by individually comparing the expression levels of the PSR genes between 6 DAA and 15 DAA.

A number of PSR genes and other genes from PT, SPX and PAP families had expression profiles consistent with involvement in the remobilization of P from senescing leaves during grain filling. One of these genes, *OsPAP26*, is the rice orthologue of *AtPAP26* which plays a role in P remobilization from senescing leaves to developing *Arabidopsis* seeds (Robinson *et al*., 2012). Interestingly, *OsPAP26* has not been implicated in plant responses to P starvation at the vegetative stage, and therefore, its role in P remobilization may be specific to leaf senescence during grain filling (Stigter and Plaxton, 2015). In contrast, the other three *OsPAP* genes with highly up‐regulated expression at 15 DAA in flag leaves, *OsPAP3c*,* OsPAP9b* and *OsPAP10a*, show increased expression in rice under P starvation at the vegetative stage (Secco *et al*., 2013; Zhang *et al*., 2011) and may have a broader role in P remobilization from leaves.

Two genes in the SPX‐MFS subfamily *OsSPX‐MFS1* and *OsSPX‐MFS2* were highly up‐regulated at 15 DAA. Wang *et al*. (2012) observed *OsSPX‐MSF1* mutant plants accumulated Pi in leaves which led them to suggest *OsSPX‐MFS1* is a P transporter in leaves during vegetative growth. Up‐regulation of these same genes during senescence is consistent with them functioning as P transporters during remobilization of Pi from leaves during senescence. *OsSPX1* and *OsSPX2* were two of the 26 PSR genes that were up‐regulated at 15 DAA, and although up‐regulated expression may have arisen in response to the reduced levels of available Pi in the leaves, the similarity in structure and expression levels to *OsSPX‐MFS1* and *OsSPX‐MFS2* suggests these genes may have been in part responsible for the reduced levels of available Pi in the leaves. While both *OsSPX‐MFS1* and *OsSPX‐MFS2* are regulated by the transcription factor *OsPHR2* under P starvation (Wang *et al*., 2012), up‐regulation of *OsSPX‐MFS1* and *OsSPX‐MFS2* at 15 DAA in the present study did not correspond to increased expression of *OsPHR2*. This suggests that, as with the regulation of the *Arabidopsis AtPAP26* (Stigter and Plaxton, 2015), key regulatory genes in the P starvation response are not necessarily involved in regulation of P remobilization from vegetative tissues during grain filling.

Three P transporters (*OsPT5, OsPT19 and OsPT20*) were up‐regulated while the expression of another four (*OsPT8*,* OsPT14, OsPT16 and OsPT22*) was down‐regulated at 15 DAA. RNAi evidence has suggested *OsPT8* is associated with P translocation from vegetative organs to reproductive tissues (Jia *et al*., 2011) which is contrary to the data presented here. A similar role has been assigned to *AtPHT2; 1*, an *Arabidopsis* orthologue of *OsPT14* that was also down‐regulated at 15 DAA. The expression level of two P transporters, *OsPT21* and *OsPT24*, were similar at the two stages although their expression was much higher than all other P transporters at both 6 DAA and 15 DAA. It has been suggested that *OsPT21*–*OsPT26* transport P between the cytosol and plastids in leaves and roots (Guo *et al*., 2008) so high expression of these genes may be indicative of the need to maintain Pi required for photosynthesis (Sivak and Walker, 1986). Despite a recent review of P remobilization during leaf senescence concluding that Pi transporters facilitate the translocation of P liberated from organic P sources to developing grain (Stigter and Plaxton, 2015), we found no evidence for up‐regulation of any of the 26 known Pi transports in rice in transport of Pi from senescing flag leaves.

Several genes that participate in dephosphorylation such as *OsPAP1d*,* OsPAP3c*,* OsPAP10c*,* OsPLDz2* and glycerophosphoryl diester phosphodiesterase and genes related to lipid metabolism, such as digalactosyldiacylglycerol (DGDG) and monogalactosyldiacylglycerol (MGDG) synthases, were up‐regulated at 15 DAA. Enhanced activity of lipid phosphatases may be related to the activity of plastid‐located MGDG and DGDG synthases for maintenance of membrane lipids in these organelles, which has been observed under low temperature stress (Campos *et al*., 2003). In particular, *OsPLDz2* has been implicated in dephosphorylation of plasma membranes and endoplasmic reticulum phospholipids, generating DAG and releasing phosphate upon P starvation (Cruz‐Ramírez *et al*., 2006) with the phosphate becoming available for translocation to the developing grains. Moreover, three genes whose products are directly related to *de novo* synthesis of DAG, glycerol 3‐phosphate dehydrogenase, dihydroxyacetone kinase and glycerol 3‐phosphate O‐acyltransferase were down‐regulated, indicating that this process is reduced during senescence and highlighting the importance of phospholipid degradation for the release of DAG and Pi. Sulphoquinovosyl diacylglycerols substitute for phosphatidylglycerol to maintain the proportion of anionic lipids under P starvation (Yu and Benning, 2003). The replacement of phospholipids with sulpholipids and galactolipids is a key strategy used by plants from the Proteaceae family to maintain photosynthesis in P‐limited environments (Lambers *et al*., 2012). These observations therefore support the notion that the genes involved in recycling P from phospholipids during P starvation in leaves during vegetative growth are also involved in recycling P from leaf phospholipids during leaf senescence which could be translocated to the filling grain. Whether the replacement of phospholipids with other lipids successfully enables flag leaves to maintain high levels of photosynthesis, as occurs when phospholipids are replaced in P‐efficient Proteaceae (Lambers *et al*., 2012), or whether there is a resultant decline in photosynthesis, is not known.

RNA‐P is a relatively large component of the metabolically active P pool in plants (Veneklaas *et al*., 2012), and it was hypothesized *RNS2* is part of the P scavenging system under P starvation stress (Abel *et al*., 2002). Shane *et al*. (2014) demonstrated RNase activity is highly up‐regulated during leaf senescence in *Hakea prostrata*, and transcriptome analyses indicated that the S‐like RNase, *AtRNS2,* plays a role in the P starvation response during vegetative growth, but is also induced by senescence in *Arabidopsis* (Hillwig *et al*., 2011; Taylor *et al*., 1993). Our results demonstrate that two genes related to RNA degradation were significantly up‐regulated at 15 DAA (log_2_ = 1.8 and 0.9; Table 4). The genes coded by Os10g0556700 and Os10g0556600 correspond to CCR4‐NOT transcription complex subunit 1, a deadenylase participating in poly (A) shortening (Temme *et al*., 2014), while Os07g0249600 codes for an mRNA‐decapping enzyme 1B (Lykke‐Andersen, 2002). These two enzymes act on two of the RNA protecting mechanisms, capping and polyadenylation, indicating that during rice leaf senescence, RNA degradation occurs leading to the release of P for its redistribution to the seeds. So far, neither of these two enzymes have been identified as induced by P starvation or leaf senescence; thus, our results widen our knowledge of alternative RNases participating in the liberation of P during leaf senescence. Interestingly, at 15 DAA, the expression of Os07g0661000 and Os04g0680400, that code for an adenosine deaminase and an allantoinase, respectively, were also up‐regulated, opening the possibility to associate the activity of the corresponding enzymes with that of CCR4‐NOT. Deadenylation by the latter would lead to an increase in cytoplasmic adenosine which would be eventually degraded by the consorted activity of adenosine deaminase and allantoinase, to finally generate CO_2_ and NH_3,_ which could be reassimilated via the glutamine oxoglutarate aminotransferase (GOGAT) pathway (Moffatt and Ashihara, 2002), indicating that nucleotide degradation also serves to recirculate N during leaf senescence.

## Conclusions

Several genes involved in the phosphate starvation response were found to have gene expression profiles consistent with a role in the remobilization of P from senescing flag leaves. While some of these genes have previously been implicated in the P starvation response, it was evident that some of these genes (e.g. *OsSPX‐MFS1* and *OsSPX‐MFS2*) are under different regulatory control during senescence. The fact that a number of genes that have not been implicated in the P starvation response were identified provides potential novel targets for manipulation to reduce the quantity of P loaded into cereal grains, thus reducing the amount of P removed from farmers' fields at harvest. Further studies are evidently needed to confirm the involvement of genes identified in the present study and to explore whether further novel genes or signalling molecules that have not been implicated in the P starvation response play a role in P remobilization from senescing leaves to developing grains.

## Experimental procedures

### Soil and plant material

Soil from the 0‐ to 10‐cm horizon was collected from Southern Cross University's Brookside field site on the Lismore campus (28°49′3.74″ S, 153°18′21.49″ E). A detailed description of the soil is given in Rose *et al*. (2015). Briefly, the soil was pH 5.81 (1:5 H_2_O); total carbon 2.21%; electrical conductivity 0.48 dS/m; Bray P 0.6 mg/kg; and effective cation exchange capacity 13.6 cmol^+^/kg. The soil was air‐dried and passed through a 2 mm sieve before being added at 8 kg/pot to 10 L, nondraining, black plastic pots. Immediately prior to transplanting rice seedlings (see below), nitrogen (N, as urea) and P (as superphosphate) were mixed into the soil for each pot at rates equivalent to 100 kg N/ha and 50 kg P/ha, respectively, on a pot soil surface area basis, before soils were flooded with tap water.

Seeds of the widely grown rice (mega) variety IR64 were sterilized using HClO_3_ for 2 min before germination in Petri dishes in the dark at 30 °C for 2 days. Germinated seeds were transferred to a mesh floating on a solution containing 0.1 mm calcium (Ca) and 36 μm iron (Fe). After 10 days, the solution was changed to half strength Yoshida solution (Yoshida *et al*., 1976) and plants were grown for another 2 weeks, with nutrient solution replaced every week. Seedlings were then transplanted into soil with three evenly sized seedlings planted in each pot.

Plants were grown under controlled glasshouse conditions at Southern Cross University (Lismore, NSW, Australia) with a mean day/night air temperature of 29 °C/21 °C and relative humidity (RH) of 75%. Additional N (50 kg/ha as urea) was top‐dressed at tillering stage (60 days after transplanting) to ensure low soil N did not induce premature leaf senescence.

Individual panicles in each pot were tagged at anthesis, with anthesis defined as when 50% of florets on a panicle had flowered. This occurred at around 85 days after sowing (DAS). At anthesis, and every 3 DAA until maturity, panicles of the same age (excluding panicles of the main tiller) from three separate pots were harvested and separated into grain, husk, rachis, stem, flag leaf and other leaves. This occurred until 33 DAA, when plants reached physiological maturity, with no plant sampled more than once. After weighing, fresh tissue samples were divided into half: one subsample was immediately frozen in liquid nitrogen and kept at −80 °C for later RNA extraction and RNA‐seq analysis, and the second subsample was dried for 7 days at 40 °C in a drying room.

### Tissue biomass and P measurements

Phosphorus concentration in flag leaf tissue was measured by digesting 0.2 g tissue with 5 mL of nitric acid (HNO_3_) in a MARS Xpress microwave oven (CEM Corporation, NC, Charlotte). After digestion, each sample was diluted by addition of purified water (Milli‐Q, Millipore, Billerica, MA, US) to a final volume of 25 mL and P concentration measured by inductively coupled plasma mass spectrometry (ICP‐MS) (Perkin Elmer, Melbourne, Victoria, Australia).

### RNA extraction and library preparation

RNA extraction was undertaken on flag leaf tissue samples from two time points selected from the P audit (above). Total RNA was extracted from rice flag leaf tissue using the RNeasy Mini Kit (Qiagen, Victoria, Australia, Chadstone) according to the manufacturer's instructions. After extraction, RNA was quantified using the Nanodrop (ND1000; Labtech, Paris, France) and RNA quality was then examined using a 2100 Bioanalyzer (Agilent Technologies, CA, Santa Clara). High‐quality RNA samples for library construction were selected based on 260/280 nm ratio and RNA integrity number (RIN) above 2.0 and 8.0, respectively. Samples that did not meet these quality criteria were re‐extracted. The TruSeq mRNA stranded kit (Illumina, Scoresby, Vicotria, Australia) was used to prepare Illumina RNA‐seq libraries for each sample from 1.3 μg of total RNA. The concentration of each library was measured by qPCR using the KAPA library quantification kit (KAPA Biosystems, MA, Wilmington) to determine the required number of PCR cycles for library amplification. The final concentration of the amplified library was measured using Qubit^®^ Fluorometric Quantitation (Life Technologies, CA, Carlsbad). Library sequencing was undertaken with the Illumina Hi‐Seq 2500 system (Illumina) at the Biomolecular Facility (BRF) at the John Curtin School for Medical Research (JCSMR), Australian National University (ANU), ACT, Australia. The raw sequencing data have been uploaded to ENA (European Nucleotide Archive) database (accession number: PRJEB11899).

### RNA‐seq data analysis

Raw sequencing reads in FASTQ format were first filtered for quality using FASTQC (Andrews, 2010) followed by removal of adapter sequences, poly‐N stretches and low‐quality reads using the BBDuk module of the BBMap software package (http://sourceforge.net/projects/bbmap/). All subsequent analyses were based on high‐quality sequencing reads. Bowtie v2.2.4 (Langmead and Salzberg, 2012) was used to index the genome. Retained high‐quality paired‐end reads were mapped against the rice genome IRGSP 1.0 using TopHat (Trapnell *et al*., 2009), which is a splice aware aligner of RNA‐Seq reads. The Ensembl Plants (http://plants.ensembl.org/Oryza_sativa/Info/Annotation) *O. sativa* cv. Nipponbare (ssp. *japonica*) reference genome annotation was utilized. TopHat identified the exon–exon junctions and produced the read vs genome alignment in BAM (Binary Alignment Map) format. Cufflinks (Trapnell *et al*., 2012) then used the TopHat‐generated alignment to assemble a set of reference‐based transcripts. Finally, the CuffDiff module of Cufflinks was used to identify differentially expressed genes between samples and CummeRbund (http://compbio.mit.edu/cummeRbund/) R package was used for subsequent analyses.

### Involvement of PSR genes in phosphorus remobilization from flag leaves during grain filling

To investigate whether any PSR genes were involved in remobilization of P from leaves during grain filling, we identified a total of 115 PSR genes, including 26 *OsPT* genes and eight *RNS* genes, based on recent literature (Lin *et al*., 2009; Liu *et al*., 2011; MacIntosh *et al*., 2010; Secco *et al*., 2013) (Table S1). First, we assessed the absolute FPKM value of the known 115 PSR genes at the two selected time points. We also compared their expression to the expression of the 30 most highly expressed genes to assess their relative level of expression. Secondly, we assessed the relative change in their expression between the two time points when the flag leaf had a stable P concentration compared with when the leaf acted as P source tissue.

Gene expression was evaluated based on FPKM values (i.e. FPKM > 0). Significant DEGs were defined based on *P* and FDR (i.e. <0.05).

### GO analysis of DEGs using RNA‐seq data

To analyse the GO enrichment analysis, firstly, significantly differential expressed genes were retrieved by cutting off based on *P* and FDR values <0.05, respectively, and log_2_ fold change >1.5. Total of 1643 DEGs were retrieved of 5160 DEGs. GO terms for each rice gene were obtained using PAGE analysis in AgriGO public web tool (http://bioinfo.cau.edu.cn/agriGO/analysis.php?method=PAGE) which can be accepted an arbitrarily large input list with fold change (Du *et al*., 2010).

### Expression of *OsPTs*,* OsPAPs* and *OsSPX* domains in the flag leaf at 6 DAA and 15 DAA

Several *OsPTs*,* OsPAPs* and *OsSPXs* were among the 115 PSR genes with differential expression between the two time points. We therefore investigated the expression of all known members of these gene families at 6 DAA and 15 DAA, including those that were not among the 115 PSR genes. This included the *OsSPX‐MFS* genes that are classified as SPX domain‐possessing proteins that are regulated by Pi starvation in rice and preferentially expressed in the leaves (Lin *et al*., 2010; Wang *et al*., 2012).

### Biological pathway analysis

We examined whether metabolic pathways that include RNase or phospholipase genes were more active when the flag leaf was acting as a P source (15 DAA) using the KEGG (Kyoto Encyclopedia of Genes and Genomes) pathway tool. We assumed that genes differentially expressed between 6 DAA and 15 DAA, particularly with elevated expression 15 DAA, might be involved in P remobilization. We therefore retrieved from the KEGG pathway database all the activating enzymes/genes related with lipid and nucleotide metabolism, such as glycerophospholipid metabolism, glycerolipid metabolism, sphingolipid metabolism, glycosphingolipid metabolism and RNA transport/degradation. Those retrieved genes were then mapped to the RNA‐seq data to verify their involvement based on log_2_ fold change and significance.

## Supporting information


**Table S1** 115 PSR *genes* identified from recent literature and their expression values between 6 DAA and 15 DAA in rice flag leaves.
