# Supplementary material for: Phosphorus remobilization from rice flag leaves during grain filling: an RNA‐seq study
Source: Plant Biotechnol J. 2016 Jun 27;15(1):15–26. doi: 10.1111/pbi.12586 (PMC5253468; doi:10.1111/pbi.12586)
Supplement: Supplementary file 1 — Table S1 115 PSR genes identified from recent literature and their expression values between 6 DAA and 15 DAA in rice flag leaves. [file PBI-15-15-s001.pdf]

Supporting information

Table S1 The list of 115 PSR genes and their expression values between two time points

No.	Gene id	MSU id	Name / Description	FPKM 6 DAA	FPKM 15 DAA	log_2	Significance*	References
1	OS01G0110100	LOC_Os01g02000	*OsPHO1;1*	2.50	7.30	1.55	yes	Secco et al. 2013
2	OS01G0142300	LOC_Os01g04920	*OsSQD2*	12.03	37.18	1.63	yes	Secco et al. 2013
3	OS01G0159800	LOC_Os01g06640	*OsbHLH035*	0.81	1.38	0.78	no	Lin et al. 2009
4	OS01G0279700	LOC_Os01g17240	*OsPT21*	1613.60	1168.25	-0.47	no	Liu et al. 2011
5	OS01G0310100	LOC_Os01g20860	*OsPLDzeta2*	3.35	8.74	1.38	yes	Secco et al. 2013
6	OS01G0557500	LOC_Os01g37690	*OsCAX1a*	19.79	38.98	0.98	yes	Lin et al. 2009
7	OS01G0657100	LOC_Os01g46860	*OsPT11*	0.03	0.02	-0.55	no	Liu et al. 2011
8	OS01G0720400	LOC_Os01g52230	*OsACP1*	6.28	9.67	0.62	no	Secco et al. 2013
9	OS01G0758300	LOC_Os01g55350	Phosphoenolpyruvate carboxylase	0.00	0.00	0.00	no	Secco et al. 2013
10	OS01G0763600	LOC_Os01g55780	Glycerophosphoryl diester phosphodiesterase family protein	19.91	23.76	0.25	no	Secco et al. 2013
11	OS01G0776600	LOC_Os01g56880	*OsPAP10a*	16.63	41.37	1.31	yes	Secco et al. 2013
12	OS01G0852200	LOC_Os01g63290	*OsPT22*	49.99	18.59	-1.43	yes	Liu et al. 2011
13	OS01G0897200	LOC_Os01g67180	*OsRNS2*	42.29	47.11	0.16	no	MachIntosh et al. 2010
14	OS01G0897300	LOC_Os01g67190	*OsRNS6*	3.52	1.82	-0.95	no	MachIntosh et al. 2010
15	OS01G0901800	LOC_Os01g67560	Divergent PAP2 family domain containing protein	6.35	8.10	0.35	no	Secco et al. 2013
16	OS01G0915000	LOC_Os01g68650	Protein of unknown function DUF506, plant family protein.	0.16	0.02	-2.77	no	Secco et al. 2013
17	OS01G0954400	LOC_Os01g72480	Zinc finger, C3HC4 type domain containing protein	41.46	36.01	-0.20	no	Secco et al. 2013
18	OS02G0202200	LOC_Os02g10780	*OsSPX2*	33.67	54.16	0.69	yes	Secco et al. 2013

Table S1 continued.

No.	Gene id	MSU id	Name / Description	FPKM		log_2	Significance*	References
				6 DAA	15 DAA			
19	OS02G0226200	LOC_Os02g13290	phosphoethanolamine/phosphocholine phosphatase	0.62	0.16	-1.96	no	Secco et al. 2013
20	OS02G0314300	LOC_Os02g21009	*OsCAX1c*	87.48	63.31	-0.47	no	Lin et al. 2009
21	OS02G0325600	LOC_Os02g22020	Similar to Phosphate starvation response regulator-like protein	0.07	0.28	1.98	no	Secco et al. 2013
22	OS02G0327000	LOC_Os02g22130	C2 domain containing protein	60.97	65.37	0.10	no	Secco et al. 2013
23	OS02G0514500	LOC_Os02g31030	Glycerophosphoryl diester phosphodiesterase family protein	6.06	24.77	2.03	yes	Secco et al. 2013
24	OS02G0593500	LOC_Os02g38020	*OsPT14*	35.75	16.15	-1.15	yes	Liu et al. 2011
25	OS02G0625300	LOC_Os02g41580	CAMK_CAMK_like.14 - CAMK includes calcium/calmodulin depedent protein kinases	38.41	25.68	-0.58	yes	Secco et al. 2013
26	OS02G0668500	LOC_Os02g44820	Cellular retinaldehyde-binding/triple function, C-terminal domain containing protein	35.42	12.24	-1.53	yes	Secco et al. 2013
27	OS02G0767500	LOC_Os02g52860	*OsPT15*	49.14	44.85	-0.13	no	Liu et al. 2011
28	OS02G0786200	LOC_Os02g54510	Protein kinase domain containing protein	40.57	52.76	0.38	no	Secco et al. 2013
29	OS02G0802700	LOC_Os02g55910	Similar to MGDG synthase type A	12.18	34.87	1.52	yes	Secco et al. 2013
30	OS02G0809800	LOC_Os02g56510	*OsPHO1;2*	0.09	0.39	2.08	no	Lin et al. 2009
31	OS03G0136400	LOC_Os03g04360	*OsPT7*	0.00	0.00	0.00	no	Liu et al. 2011
32	OS03G0150500	LOC_Os03g05610	*OsPT12*	0.00	0.00	0.00	no	Liu et al. 2011
33	OS03G0150600	LOC_Os03g05620	*OsPT1*	40.78	32.74	-0.32	no	Liu et al. 2011
34	OS03G0150800	LOC_Os03g05640	*OsPT2*	0.11	0.00	Undefined$	no	Liu et al. 2011
35	OS03G0214400	LOC_Os03g11560	Digalactosyldiacylglycerol synthase, chloroplast precursor	5.74	11.04	0.94	yes	Secco et al. 2013
36	OS03G0238600	LOC_Os03g13540	*OsPAP3c*	10.74	59.36	2.47	yes	Secco et al. 2013

Table S1 continued.

No.	Gene id	MSU id	Name / Description	FPKM		log_2	Significance*	References
				6 DAA	15 DAA			
37	OS03G0261800	LOC_Os03g15530	Protein of unknown function DUF3049 domain containing protein	12.71	33.58	1.40	yes	Secco et al. 2013
38	OS03G0263400	LOC_Os03g15690	*OsPT16*	11.83	5.05	-1.23	yes	Liu et al. 2011
39	OS03G0267700	LOC_Os03g16080	mitochondrial carrier protein	17.91	19.16	0.10	no	Secco et al. 2013
40	OS03G0329900	LOC_Os03g21240	*OsPHR1*	41.51	37.68	-0.14	no	Lin et al. 2009
41	OS03G0406100	LOC_Os03g29250	*OsSPX5*	0.00	0.24	Undefined	no	Secco et al. 2013
42	OS03G0587000	LOC_Os03g39000	inositol-1-monophosphatase, Similar to L-galactose-1-phosphate phosphatase	25.54	18.87	-0.44	no	Secco et al. 2013
43	OS03G0603600	LOC_Os03g40670	Glycerophosphoryl diester phosphodiesterase family protein	1.39	1.44	0.05	no	Secco et al. 2013
44	OS03G0647600	LOC_Os03g44540	Nuclear transcription factor Y subunit	4.56	4.00	-0.19	no	Secco et al. 2013
45	OS03G0719100	LOC_Os03g50980	*OsSIZ2*	22.15	21.66	-0.03	no	Lin et al. 2009
46	OS03G0852800	LOC_Os03g63580	Phosphoesterase family protein	0.02	0.20	3.46	no	Secco et al. 2013
47	OS04G0185600	LOC_Os04g10690	*OsPT5*	0.30	0.90	1.60	yes	Liu et al. 2011
48	OS04G0186400	LOC_Os04g10750	*OsPT4*	1.11	2.42	1.12	no	Liu et al. 2011, Secco et al. 2013
49	OS04G0186800	LOC_Os04g10800	*OsPT13*	0.01	0.03	1.06	no	Liu et al. 2011
50	OS04G0448800	LOC_Os04g37600	*OsPT17*	16.09	11.78	-0.45	no	Liu et al. 2011
51	OS04G0555300	LOC_Os04g46880	Similar to glycerol 3-phosphate permease.	5.85	10.13	0.79	no	Secco et al. 2013
52	OS04G0561200	LOC_Os04g47330	rho-GTPase-activating protein-related	1.36	0.23	-2.55	no	Secco et al. 2013
53	OS04G0598000	LOC_Os04g50970	Seed specific protein Bn15D1B	553.88	297.77	-0.90	yes	Secco et al. 2013
54	OS04G0608600	LOC_Os04g51920	Protein disulfide isomerase	14.28	9.11	-0.65	yes	Secco et al. 2013

Table S1 continued.

No.	Gene id	MSU id	Name / Description	FPKM		log_2	Significance *	References
				6 DAA	15 DAA			
55	OS04G0631100	LOC_Os04g53930	Organic cation transporter protein	0.66	0.28	-1.25	no	Secco et al. 2013
56	OS04G0661200	LOC_Os04g56580	*OsIPK1*	3.18	3.62	0.19	no	Lin et al. 2009
57	OS05G0114000	LOC_Os05g02310	Soluble inorganic pyrophosphatase	1.54	2.93	0.93	no	Secco et al. 2013
58	OS05G0125000	LOC_Os05g03430	*OsSIZ1*	25.64	28.06	0.13	no	Lin et al. 2009
59	OS05G0358700	LOC_Os05g29050	*OsPLDrho1*	9.97	16.72	0.75	yes	Lin et al. 2009
60	OS05G0387200	LOC_Os05g32140	Similar to UDP-sulfoquinovose synthase, chloroplast precursor (EC 3.13.1.1)	219.30	60.06	-1.87	yes	Secco et al. 2013
61	OS05G0451100	LOC_Os05g37820	*OsPT23*	3.43	1.68	-1.03	no	Liu et al. 2011
62	OS05G0468600	LOC_Os05g39230	Low photochemical bleaching 1 protein	0.00	0.00	0.00	no	Secco et al. 2013
63	OS05G0557700	LOC_Os05g48390	*OsPHO2*	8.93	18.89	1.08	yes	Lin et al. 2009
64	OS06G0140800	LOC_Os06g04880	Serine threonine kinase	24.09	39.60	0.72	yes	Secco et al. 2013
65	OS06G0193400	LOC_Os06g09370	*OsPTF1, OsbHLH096*	38.52	46.15	0.26	no	Lin et al. 2009
66	OS06G0208700	LOC_Os06g10650	Tyrosine phosphatase family protein	3.78	3.49	-0.11	no	Secco et al. 2013
67	OS06G0210500	LOC_Os06g10810	*OsPT18*	11.30	13.76	0.28	no	Liu et al. 2011
68	OS06G0291500	LOC_Os06g18820	Conserved hypothetical protein.	0.34	1.36	1.99	yes	Secco et al. 2013
69	OS06G0324800	LOC_Os06g21920	*OsPT9*	0.02	0.00	Undefined	no	Liu et al. 2011
70	OS06G0325200	LOC_Os06g21950	*OsPT10*	0.00	0.00	0.00	no	Liu et al. 2011, Secco et al. 2013
71	OS06G0493600	LOC_Os06g29790	*OsPHO1;3, PHo1;3*	8.00	12.52	0.65	no	Lin et al. 2009, Secco et al. 2013
72	OS06G0603600	LOC_Os06g40120	*OsSPX1*	31.50	51.15	0.70	yes	Lin et al. 2009, Secco et al. 2013

Table S1 continued.

No.	Gene id	MSU id	Name / Description	FPKM 6 DAA	FPKM 15 DAA	log_2	Significance[*]	References
73	OS06G0697000	LOC_Os06g48200	Glycosyl hydrolases family 16	0.14	0.29	1.01	no	Secco et al. 2013
74	OS06G0730300	LOC_Os06g51390	Protein of unknown function DUF829	69.09	75.46	0.13	no	Secco et al. 2013
75	OS07G0100300	LOC_Os07g01030	Glycosyl transferase, group 1 domain containing protein	131.17	80.56	-0.70	yes	Secco et al. 2013
76	OS07G0106000	LOC_Os07g01540	Metallophosphoesterase domain containing protein	7.27	9.23	0.34	no	Secco et al. 2013
77	OS07G0134500	LOC_Os07g04210	Similar to hydrolase/ protein serine/threonine phosphatase	9.82	20.74	1.08	yes	Secco et al. 2013
78	OS07G0165200	LOC_Os07g07080	Regulator of chromosome condensation/beta-lactamase-inhibitor protein II	8.59	35.30	2.04	yes	Secco et al. 2013
79	OS07G0187400	LOC_Os07g08970	Conserved hypothetical protein	44.01	288.27	2.71	yes	Secco et al. 2013
80	OS07G0187700	LOC_Os07g09000	*OsPHF1*	28.30	12.13	-1.22	yes	Lin et al. 2009, Secco et al. 2013
81	OS07G0438800	LOC_Os07g25710	*OsPHR2*	61.15	56.60	-0.11	no	Lin et al. 2009
82	OS07G0500300	LOC_Os07g31720	C2 calcium-dependent membrane targeting domain containing protein	22.38	17.72	-0.34	no	Secco et al. 2013
83	OS07G0588000	LOC_Os07g39900	Interferon-related developmental regulator domain containing protein	67.26	78.72	0.23	no	Secco et al. 2013
84	OS07G0629300	LOC_Os07g43600	*OsRNS7*	0.00	0.00	0.00	no	MachIntosh et al. 2010
85	OS07G0629900	LOC_Os07g43640	*OsRNS8*	0.00	0.00	0.00	no	MachIntosh et al. 2010
86	OS07G0630400	LOC_Os07g43670	*OsRNS1*	0.00	0.00	0.00	no	MachIntosh et al. 2010, Secco et al. 2013
87	OS08G0156600	LOC_Os08g06010	Major facilitator superfamily protein.	8.95	18.34	1.03	yes	Secco et al. 2013
88	OS08G0280100	LOC_Os08g17784	*OsPAP23*	0.37	0.54	0.55	no	Secco et al. 2013
89	OS08G0299400	LOC_Os08g20420	Similar to MGDG synthase type A.	0.02	0.22	3.70	no	Secco et al. 2013
90	OS08G0433200	LOC_Os08g33640	Conserved hypothetical protein.	34.26	50.60	0.56	yes	Secco et al. 2013

Table S1 continued.

No.	Gene id	MSU id	Name / Description	FPKM		log_2	Significance*	References
				6 DAA	15 DAA			
91	OS08G0434100	LOC_Os08g33710	*OsRNS3*	0.56	1.13	1.01	no	MachIntosh et al. 2010, Secco et al. 2013
92	OS08G0535700	LOC_Os08g42390	glycerophosphoryl diester phosphodiesterase family protein	4.30	2.20	-0.96	no	Secco et al. 2013
93	OS08G0564000	LOC_Os08g45000	*OsPT6*	0.00	0.00	0.00	no	Liu et al. 2011
94	OS09G0315700	LOC_Os09g14670	Phosphoenolpyruvate carboxylase	2.27	2.72	0.26	no	Secco et al. 2013
95	OS09G0407700	LOC_Os09g24230	Haloacid dehalogenase-like hydrolase domain containing protein	16.03	21.66	0.43	no	Secco et al. 2013
96	OS09G0438100	LOC_Os09g26670	Conserved hypothetical protein.	0.13	0.44	1.81	no	Secco et al. 2013
97	OS09G0454600	LOC_Os09g28160	*OsPT19*	3.56	6.68	0.91	yes	Liu et al. 2011
98	OS09G0528700	LOC_Os09g35940	Similar to Cytochrome p450 (CYP78A9)	15.78	26.39	0.74	yes	Secco et al. 2013
99	OS09G0537700	LOC_Os09g36680	*OSRNS4*	0.72	0.00	Undefined	yes	MachIntosh et al. 2010, Secco et al. 2013
100	OS09G0538000	LOC_Os09g36700	*OsRNS5*	0.00	0.00	0.00	no	MachIntosh et al. 2010
101	OS09G0554000	LOC_Os09g38100	*OsPT20*	1.80	5.00	1.47	yes	Liu et al. 2011
102	OS09G0570400	LOC_Os09g39680	*OsPT24*	694.99	334.78	-1.05	no	Liu et al. 2011
103	OS10G0116800	LOC_Os10g02750	*OsPAP3b*	0.00	0.00	0.00	no	Secco et al. 2013
104	OS10G0392600	LOC_Os10g25310	*OsSPX3*	0.00	0.00	0.00	no	Lin et al. 2009, Secco et al. 2013
105	OS10G0444600	LOC_Os10g30770	*OsPT3*	0.00	0.00	0.00	no	Liu et al. 2011
106	OS10G0444700	LOC_Os10g30790	*OsPT8*	13.26	7.11	-0.90	yes	Liu et al. 2011
107	OS11G0148000	LOC_Os11g05070	*OsCAX8*	0.70	0.88	0.33	no	Lin et al. 2009
108	OS11G0151700	LOC_Os11g05400	*OSPAP21b*	30.08	28.43	-0.08	no	Secco et al. 2013

Table S1 continued.

No.	Gene id	MSU id	Name / Description	FPKM 6 DAA	FPKM 15 DAA	log_2	Significance*	References
109	OS11G0186800	LOC_Os11g08370	*OsPT25*	1.26	1.01	-0.32	no	Liu et al. 2011
110	OS11G0236100	LOC_Os11g12810	Sucrose-phosphate synthase, putative	100.86	100.53	0.00	no	Secco et al. 2013
111	OS11G0549615	LOC_Os11g34710	*OsPAP3a*	0.05	0.00	Undefined	no	Secco et al. 2013
112	OS11G0593000	LOC_Os11g38050	Phosphoesterase family protein	0.00	0.00	0.00	no	Secco et al. 2013
113	OS12G0180100	LOC_Os12g07970	*OsPT26*	0.52	0.16	-1.71	no	Liu et al. 2011
114	OS12G0576600	LOC_Os12g38750	*OsPAP1d*	12.11	18.76	0.63	yes	Secco et al. 2013
115	OS12G0637100	LOC_Os12g44020	*OsPAP10c*	5.35	3.09	-0.79	no	Secco et al. 2013

* Significance : "Yes" indicates significantly difference between 6 DAA and 15 DAA value (P < 0.05, FDR < 0.05), "No" indicates not significant

[$] Undefined is when we have zero expression value for one of the two samples.
